# The effect of lead exposure on IQ test scores in children under 12 years: a systematic review and meta-analysis of case-control studies

**DOI:** 10.1186/s13643-022-01963-y

**Published:** 2022-05-30

**Authors:** Serve Heidari, Shayan Mostafaei, Nazanin Razazian, Mojgan Rajati, Anahita Saeedi, Fatemeh Rajati

**Affiliations:** 1 Department of Biostatistics, Faculty of Paramedical Sciences, Shahid Beheshti University of Medical Sciences, Tehran, Iran; 2 Division of Clinical Geriatrics, Department of Neurobiology, Care Sciences and Society, Karolinska Institute, Stockholm, Sweden; 3 Department of Neurology, School of Medicine, Imam Reza Hospital, Kermanshah University of Medical Sciences, Kermanshah, Iran; 4 Department of Obstetrics and Gynecology, School of Medicine Motazedi Hospital, Kermanshah University of Medical Sciences, Kermanshah, Iran; 5 Department of Biostatistics, School of Public Health & Health Sciences, University of Massachusetts, Amherst, MA USA; 6 Health Education and Promotion Department, School of Health, Research Center for Environmental Determinants of Health, Health Institute, Kermanshah University of Medical Sciences, Kermanshah, Iran

**Keywords:** Systematic review, Meta-analysis, Lead, Children, Intelligence Tests

## Abstract

An inevitable exposure to the toxic heavy metal such as lead in our environmental can have irreversible effects on children’s mental performance.

In this study, 3316 children in 8 case-control studies were selected for review. The case group was exposed to a concentration of lead above 10 μg/dL, and the control group was exposed to a concentration of less than 10 μg/dL, but the duration of exposure was different among studies, and the subgroup analysis was performed based on this variable.

In the subgroup with duration of exposure less than the average of 4.5 years, the difference of IQ test score between two groups was significant (*MD* = −3.53) (*P*-value <0.05). Also, in the subgroup with more than 4.5 years of duration, the difference of IQ test score was significant (*MD* = −22.63) (*P*-value < 0.001).

This study demonstrates that the concentration and duration of lead exposure have a large effect on mental function in children.

## Introduction

Lead is found naturally in the earth’s crust and is a toxic metal [1]. Today, its excessive use has led to its spread in the human environment, and its excessive exposure causes many problems in public health [2]. Unfortunately, in many countries, the extraction, smelting and production of lead still exist, and it is used in painting of houses, gasoline, aircraft fuel, stained glass, crystal containers, some cosmetics and toys [3]. In many cities, lead solder is used in the municipal water piping system which contaminates drinking water [4].

Young children are especially vulnerable to the toxic effects of lead [5]. Exposure to this substance has an effect on the development of the brain and the nervous system [6]. Lead also causes long-term damage in adults [7]. Lead exposure in pregnant women can cause miscarriage, stillbirth, preterm birth, and low birth weight [8]. There have been many studies on the dangers of exposure to this substance in children and adults, but because childhood is more important in the mental and brain development of individuals, it is essential to study the exposure to this substance during childhood [9].

Young children absorb 4–5 times more swallowed lead from a certain source compared to adults [10]. In addition, children’s instinctive curiosity and hand-to-mouth behavior in exploring and swallowing objects containing lead or lead coating can be dangerous [11]. Exposure to lead-contaminated soil and dust from recycling and extraction of batteries has caused mass poisoning and numerous deaths of young children in Nigeria, Senegal, and other countries [12].

High levels of lead exposure attack the brain and central nervous system, causing coma, seizures, and even death [13]. Lower levels of lead exposure, which has no obvious symptoms, lead to a range of damages to various systems in the body [14]. Lead in particular can affect children’s brain development, resulting in declined IQ level, behavioral changes such as diminished attention span, increased antisocial behavior, and decreased educational level [5].

No “safe” blood lead concentrations are known [15]. Even a blood lead concentration of 5 μg/dL may be associated with decreased intelligence in children, behavioral problems, and learning difficulties [16]. As lead exposure increases, the range and severity of symptoms and effects increase as well [17]. There have been many studies on the effects of lead exposure on children’s mental performance, such as a study by Todd et al. in 2012 in New York, who found that lead exposure at concentrations above 5 μg/dL reduced the IQ score in children by 4.9 [18]. Another study by Surkan et al. in Massachusetts revealed that at a concentration of 5–10 μg/dL, the IQ score of children in the exposure group was 7.8 points lower than in the control group, while at concentrations of 1–2 μg/dL, such a thing was not observed [19]. A 2006 study by Kordas et al. in Mexico indicated that concentrations above 10 μg/dL had a greater effect on children’s IQ scores compared to concentrations below 10 μg/dL [20]; however, Jedrychowski et al. in the USA in 2009 showed that even at very low concentrations below 5 μg/dL, the impacts of this toxin on reducing mental function in children could be observed [21].

Since an appropriate criterion for a safe dosage of lead exposure is not yet available, further investigations in this field seem necessary [22]. So far, no systematic review and meta-analysis study have been performed on children exposed to this heavy metal and its impact on their IQ among case-control studies. In this review, we aimed to estimate the effect of lead on chilren's intelligence test at different concentrations and duration of the exposure using the difference between the mean IQ scores in the exposure and control groups in children under 12 years.

##  Methods

This review included case-control studies that are divided into groups exposed to lead greater than 10 μg/dL and lower than 10 μg/dL because in these studies lead concentration upper than 10 μg/dL had been reported dangerous for brain function, and this research consists of English language articles published since December 30, 2000, to December 30, 2020. The study population included children under the age of 12 years whose duration of exposure was measured. Ravens standard progressive matrices [23], Wechsler Preschool and Primary Scale [24], Graphic Test of Reasoning (GTR) [25], Beery-Buktenica Developmental Test of Visual-Motor Integration (VMI) defined as the coordination of fine motor and visual perceptual abilities, Saudi-Based standard scores, and TONI Saudi-based scores and rank percentile were applied in the studies for measuring the IQ [26]. The possible score of IQ measurements for determining brain intelligence function was almost the same in these different scales. Therefore, the measured IQ scores did not need to be converted into a unit of measurement for conducting meta-analysis.

### Search strategies

Based on the research strategy of this study, searches were performed in PubMed, Scopus, Embase, and Cochrane Library databases. We also searched the Google Scholar for detecting the gray literature. Keywords including “intelligence,” “cognition,” “cognitive function,” “cognitive disorder,” “cognitive impairment,” “cognitive decline,” “cognitive process,” “learning,” “mental process,” “lead” “child,” “children,” “childhood,” and “kid” and their synonym were used. We also used the related MeSH and Emtree term through the PubMed and Embase, respectively. The Boolean and abbreviated expressions specific to each database were applied to combine the keywords.

The search was finally completed on December 30, 2020, and all articles were sent to EndNote X8 software. Those that did not meet the inclusion criteria were removed. The articles were first reviewed by title, and after discarding a large number of articles in this stage, the remaining articles were reviewed based on their abstracts, and again, a large number of articles were excluded due to lack of necessary criteria, and those that remained were reviewed by full text. Finally, manually searching references of included studies was used. In addition, searches were performed on articles that cited are to the included studies. All of these stages were performed by two reviewers independently, and then a third reviewer supervised their work.

Here, those studies with an exposure and a control group were included. Variables such as the concentration of lead in children’s blood test, fruits and vegetables, and also the duration of exposure to lead, were extracted. The difference between the mean scores (MD) of the IQ test in two groups of exposure and control is considered as the response variable/effect size.

The Comprehensive Meta-Analysis V2 and STATA 15 software were used for analysis. The effect size was the difference in the mean score of the IQ test between children who have been exposed to lead for more than 10 μg/dL for some times and children who have been exposed to lead less than this amount. The funnel plot and Egger’s regression test were used to examine publication bias.

## Results

**Fig. 1:**
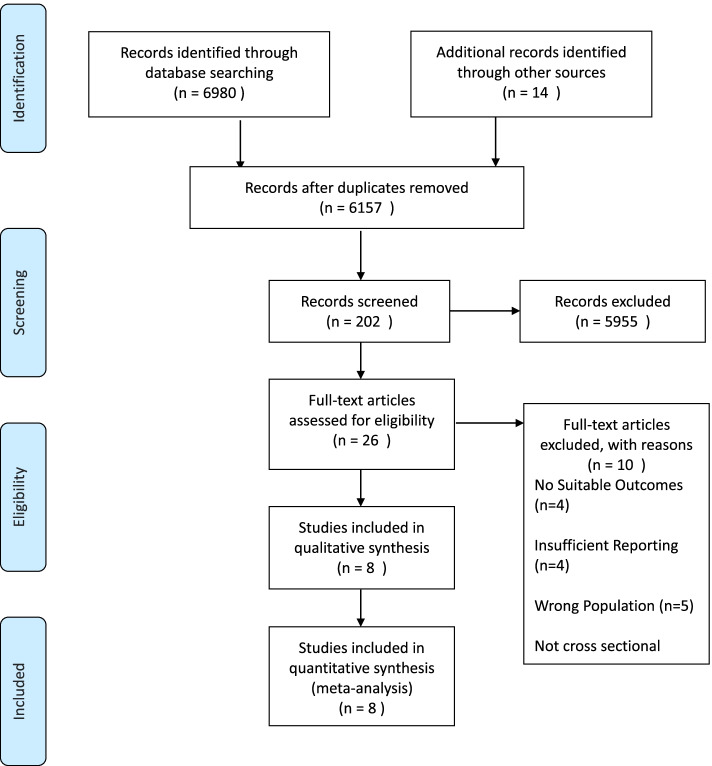
PRISMA flowchart for study selection

Systematic search results are shown in Fig. 1. In a total of 6980 articles, excluding duplicates from all site searches, 6157 articles remained. Eleven articles were obtained through manual searches in meta-analysis databases. The remaining articles were screened which was performed by two researchers to prevent errors. A total of 202 articles remained after this stage, which were reviewed based on the abstracts, and the remaining 26 articles were reviewed based on full text, and finally 8 articles remained relevant and eligible to review.

The characteristics of these 8 articles are shown in Table 1. As can be seen, the names of the authors, year of publication, country, number of samples in exposure and control groups, and the mean and standard deviation of IQ test score in each group in addition to duration of exposure and type of IQ test in different studies was extracted from included studies. The Newcastle-Ottawa Scale (NOS) scores was used for assessing the quality of case-control studies and the result of this tool is also shown in Table 1.

**Table 1:** Characteristics of the included studies

Author	Year	Country	Number of case group	Number of control group	Mean of IQ score in case group	Standard deviation of case group	Mean of IQ score in control group	Standard deviation of control group	Duration of the lead exposure (year)	Cognitive function measurement	Quality assessment (NOS)
Ibiwari C. Dike	2020	Nigeria	160	40	89.46	13.6	138.24	14.21	1.5	Raven’s standard progressive matrices	Good
Nnenna Linda Nwobi	2019	Nigeria	169	140	86.9	11.6	91.5	14	4	Raven’s standard progressive matrices	Fair
Todd A. Jusko	2008	USA	78	96	91.47	12.6	93.9	14.4	9	Wechsler Preschool and Primary Scale	Good
Pamela J. Surkan	2007	USA	32	357	88.75	12.7	91.92	10.4	4	Wechsler Intelligence Scale for Children -Third Edition (WISC-III)	Good
Katarzyna Kordas	2006	Mexico City	296	293	101.95	15.75	106.4	14.5	3	The testing battery	Fair
Katarzyna Kordas	2004	Mexico City	291	298	103.13	15.06	106.3	14.6	3	Graphic Test of Reasoning (GTR)	Good
Iman Al-Saleh 1	2001	Saudi Arabia	165	368	95.26	14.1	101.39	14.18	6	Beery VMI Saudi-based standard scores	Good
Iman Al-Saleh 2	2001	Saudi Arabia	165	368	93.51	14.61	101.37	15.09	6	TONI Saudi-based scores and rank percentile	Good

**Fig. 2:**
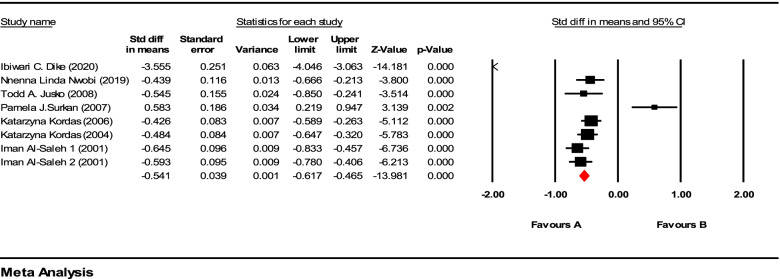
Forest plot of total analysis

Since the duration of exposure, that is the duration of the exposure to lead from birth to the time that the concentration was measured using a blood test, was diverse in different studies, we performed a total and a subgroup analysis based on duration of lead exposure, accordingly. The results of the meta-analysis totally and after performing the subgroup analysis based on the “duration of exposure” by groups are shown in Fig. 2. The average of years of exposure to lead from birth to the time of measurement was considered as cutoff point that was 4.5 years. According to Fig. 2, the effect of lead exposure on children’s IQ was significant in total (*P*-value < 0.001 and *Z*-value = −13.99).

Based on the subgroup analysis, we observe the group that was exposed to lead more than 4.5 years which the difference in the average IQ score between the group that was exposed to lead more than 10 μg/dL and the group exposed to lead less than 10 μg/dL was −22.63(95% CI:−38.69, −6.57, *P*-value < 0.001), which indicates that the IQ score in the case group was 23.30 lower than the control group, but this difference in the second subgroup, which is the subgroup that has been exposed to lead for less than 4.5 years, between the case and control groups was −3.53 (95% CI:−14.97, −7.91) that was significant (*P*-value <0.05 and *Z*-value = −2.32). As illustrated, there is a considerable difference between the two groups in terms of the mean difference based on duration of exposure.

**Fig. 3:**
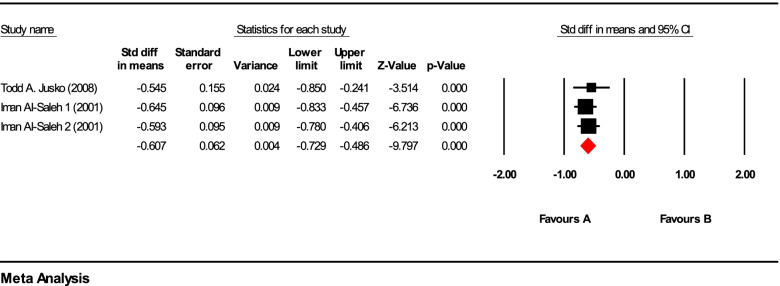
Forest plot of duration of lead exposure more than 4.5 years

**Fig. 4:**
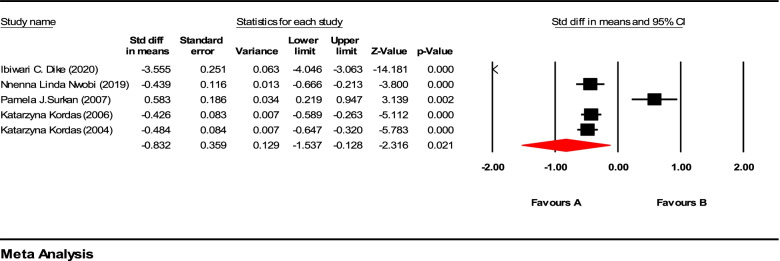
Forest plot of duration of lead exposure less than 4.5 years

Heterogeneity was generally significant with (*I*^2^ = 97.32%, *P*-value < 0.001). Heterogeneity is not observed in the first group (Fig. 3) (*I*^2^ = 0.0%, *P*-value = 1.00). However, in the second group (Fig. 4), there is a significant heterogeneity in the model (*I*^2^ = 67.42%, *P*-value = 0.048).

**Fig. 5:**
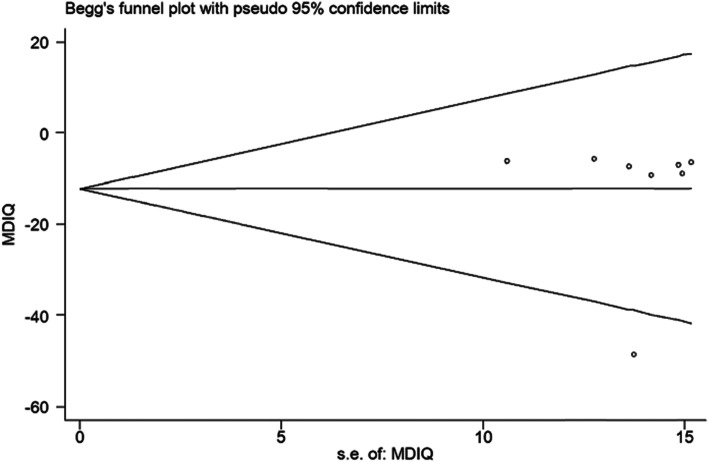
Funnel diagram of publication bias

The risk of publication bias is investigated by Egger regression, and the funnel plot is shown in Fig. 3. Based on the result of Egger test, a non-significant publication bias is seen in the model (*t* = −0.32; *P* = 0.76) (Fig. 5).

## Discussion

In the present meta-analysis, the effect of exposure to lead toxic metal in children aged fewer than 12 and its effect on their IQ score in various studies were investigated. In this paper, the difference between the mean score of IQ test was used as an effect index, and since in each study the target population had a different duration of exposure, a subgroup analysis was performed based on this variable. A total of 8 case-control studies were selected for the analysis in which the lead concentration was measured either by blood tests or by measuring lead in water and soil or in fruits and vegetables. In each study, the case group was exposed to lead concentrations above 10 μg/dL, and the control group was exposed to lead concentrations below 10 μg/dL. Different IQ tests were applied in various studies, but since all tests had almost similar band of scores, the type of test variable was not included in the subgroup analysis, and the studies were divided into two subgroups based on the duration of exposure. But, at first, analysis was done based on all studies, and then the studies with duration of exposure more than 4.5 years, which was the mean duration of exposure, were included in the first group, and studies with the duration of exposure less than 4.5 years were included in the second group. The results of the subgroup analysis suggested that the grouping was done correctly; seeing that in the first group, the difference between the mean IQ score was 22.63, and in the second subgroup, it was 3.53 which indicate a large distinction between the subgroups. A little significant heterogeneity was observed, and all studies reported reduced IQ scores at higher doses and longer duration of exposure.

According to a study in 2004 by Katarzyna Kordasa et al. on children exposed to lead, the higher the concentration of lead in the blood, the lower the IQ score of children. At concentrations above 10 μg/dL, the IQ score was much lower than at concentrations below 10 μg/dL [27]. In another study performed at a concentration of 10 μg/dL compared to 5 μg/dL, the IQ score in the case group (85.9 11 ± 11.6) and in the control group (91.5 ±14.0) showed a higher IQ score for lower concentrations [28]. In another study based on the Beery VMI Saudi-based standard scores test, it was found that at concentrations below 10 μg/dL, the IQ score was 101.39 ± 14.18, while at concentrations above 20 μg/dL, the IQ score decreased to 85.14 ± 12.94 [29]. In general, all studies are consistent in describing the toxicity of lead metal and its effect on children’s IQ.

So far, other meta-analyses have been performed to investigate the effect of lead on adults [30]; however, we conducted two meta-analyses in this topic. In our previous study, 16 studies with cohort design are included and analyzed according to “duration of exposure” and “dose” using k-means partitioning clustering algorithm. The effect size in that study was r coefficient between the cluster factors of both duration and dose exposure and variation of IQ test score (*r* = −0.22, *P*-value < 0.001) [31]. In the current study, we included the case-control studies with effect size of mean difference of IQ test scores between two groups in the meta-analysis. Therefore, these two studies are the first meta-analyses that examine the effect of lead on IQ score in children, and considering the significance of childhood as a very important period in developing mental and intellectual activities of human beings, it is very important to study the destructive effects of external harmful factors such as heavy metals like lead. It is vital to study the concentration and duration of exposure as influential factors in the effect of this toxic metal on IQ, since it helps in dealing with such a harmful factor properly. In this paper, case-control studies were used, and the difference in the mean IQ score between the two groups was considered as an effect index to examine the differences between the studies more clearly. Those articles that were not in the category of case-control studies and had different effect indices were inevitably excluded from the review cycle, and the exact duration of exposure was not available in some studies. These are the limitations of this work.

Also, the issue of determining the dose or concentration of the exposure was one of the limitations of the baseline studies, which in turn extended to this research. As it was very difficult for primary studies to consider the exact dose of the average lead exposure from baseline to blood test time, they confined the concentration of blood sample and duration of the exposure. However, primary studies discussed that the population included children from birth to the blood test time lived close to a large source of the lead, which included a factory or a mine. However, the lead concentration from blood samples will reflect an acceptable estimation of cumulative lead exposure because children lived on the same place all times. Therefore, there are some limitations in this way, which definitely go back to the baseline studies, which are explicitly mentioned in those studies.

## Conclusions

Overall, this study can be considered as evidence for the effect of lead exposure on mental and intellectual functionality during childhood. Because of some methodological issue to assess the concentration of lead exposure in total body, the result of this meta-analysis should be interpreted in caution. Since childhood is a critical period for the development of the brain during the life span, attention to the harmfulness of lead should be taken into account as much as possible. In this investigation, some studies were used, but more studies in this field should also be examined for obtaining more accurate information.
